# Supplementary material for: Spatial structure affects phage efficacy in infecting dual-strain biofilms of Pseudomonas aeruginosa
Source: Commun Biol. 2019 Nov 4;2:405. doi: 10.1038/s42003-019-0633-x (PMC6828766; doi:10.1038/s42003-019-0633-x)
Supplement: Supplementary file 1 — Supplementary Information [file 42003_2019_633_MOESM1_ESM.pdf]

Supplementary information

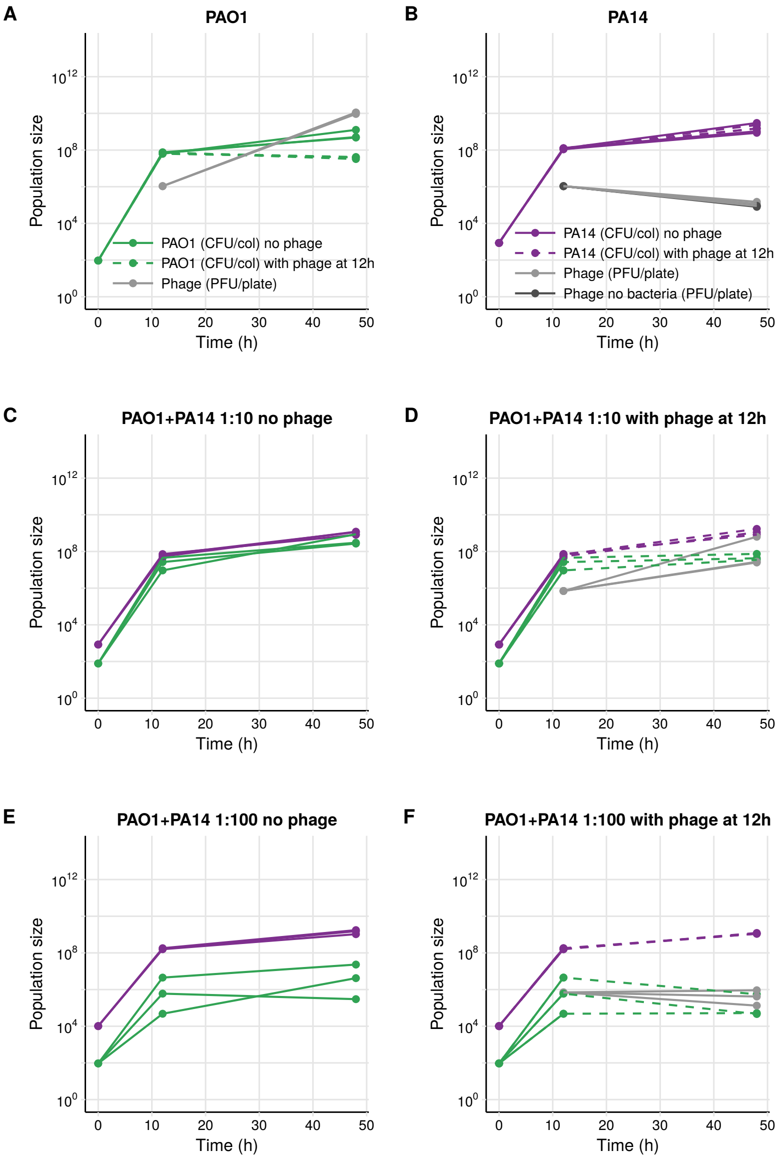

Supplementary Figure 1. Growth curves corresponding to data in Fig. 2C-F. (A) Growth curve of PAO1 with and without phage. PAO1 cease to grow following phage exposure. (B) PA14 with and without phage. No difference was observed following phage treatment, and phage decreased as in a control experiment with no bacteria. (C) Co-culture colony of the two strains at a 1:10 ratio, in the absence of phage. (D) Co-culture colony of the two strains at a 1:10 ratio, with phage, PAO1 again ceases to grow significantly. (E) Same as (C) but with a 1:100 ratio between the two strains (higher initial population size of PA14). Due to the increased competition, PAO1 cannot grow as well. (F) Same as (D) but with a 1:100 ratio between the two strains. Again, due to increased competition, there are fewer PAO1 cells, so phage cannot replicate as much. This leads to the lower overall phage population size as shown in Fig. 2F.

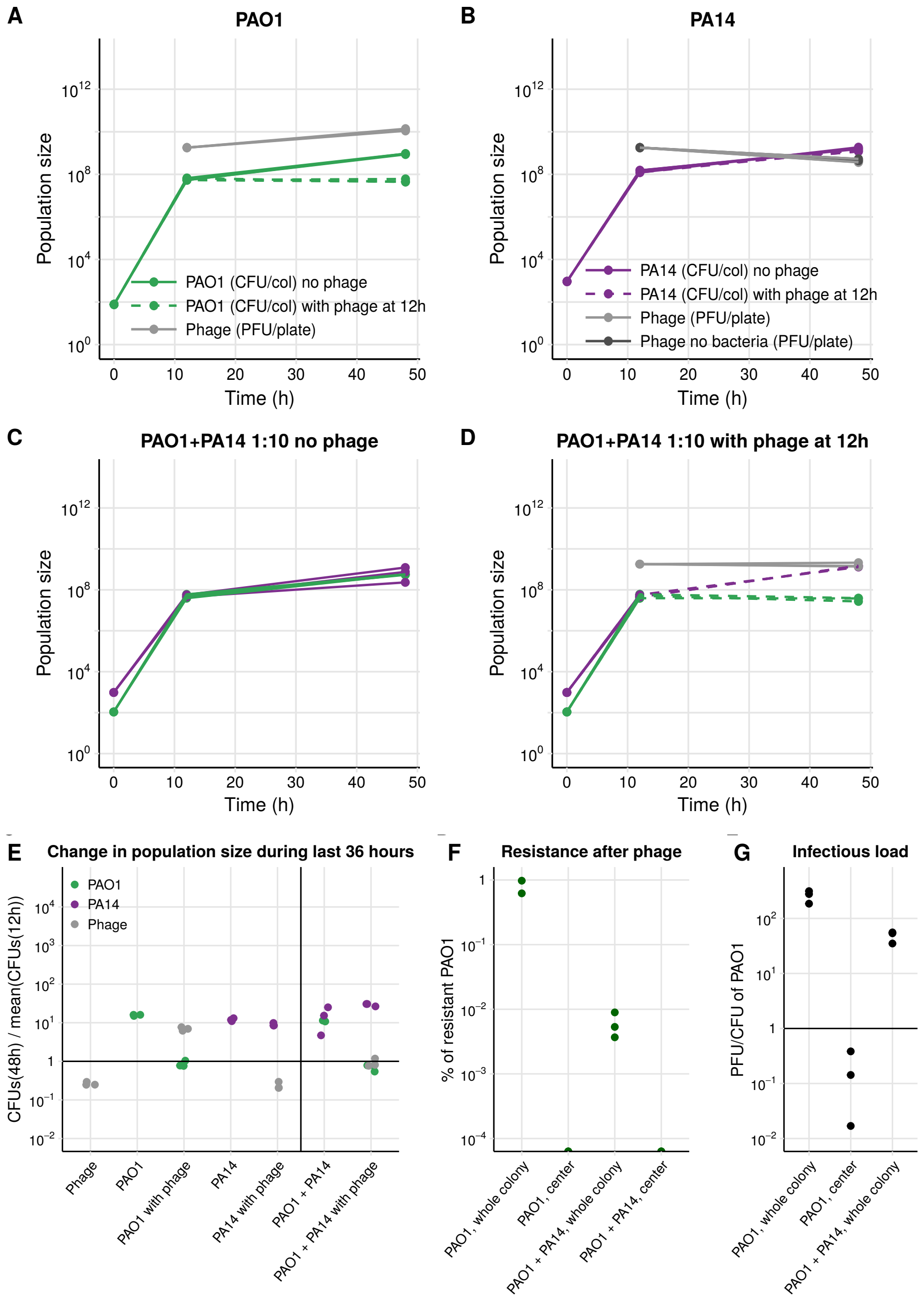

Supplementary Figure 2. Growth curves corresponding to data in Supplementary Figure 1 and 2C-F, but with an initial phage inoculum of ~10^9/ml. (A) Growth curve of PAO1 with and without phage. PAO1 cease to grow following phage exposure. (B) PA14 with and without phage. No difference was observed following phage treatment, and phage decreased as in a control experiment with no bacteria. (C) Co-culture colony of the two strains at a 1:10 ratio, in the absence of phage. (D) Co-culture colony of the two strains at a 1:10 ratio, with phage, PAO1 again ceases to grow significantly, but this time the phage do not increase significantly, probably because the population size is already very large. (E) Corresponding panel to Fig. 2C. The main difference is the lack of increase of the phage population. (F) Corresponding panel to Fig. 2D. Resistance to the phage was significantly higher in the mono-culture colonies here, but the center still only contained sensitive cells. Furthermore, although it appears that all cells were resistant, we picked 19 of the CFUs from the phage-free agar plates to test for phage resistance (see Methods), and found that at least 5 out of 19 proved to still be sensitive to the phage. In the co-culture colonies, we now detect resistance at the edges. Presumably, more phage poses a greater selection pressure leading to increased resistance. However, it is still clear that resistance is less likely in the mixed colonies. (G) To determine whether phage could diffuse into the colonies, we touched the centers with an inoculation loop and counted the PAO1 CFUs and phage PFUs. Results are comparable to Fig. 2E.

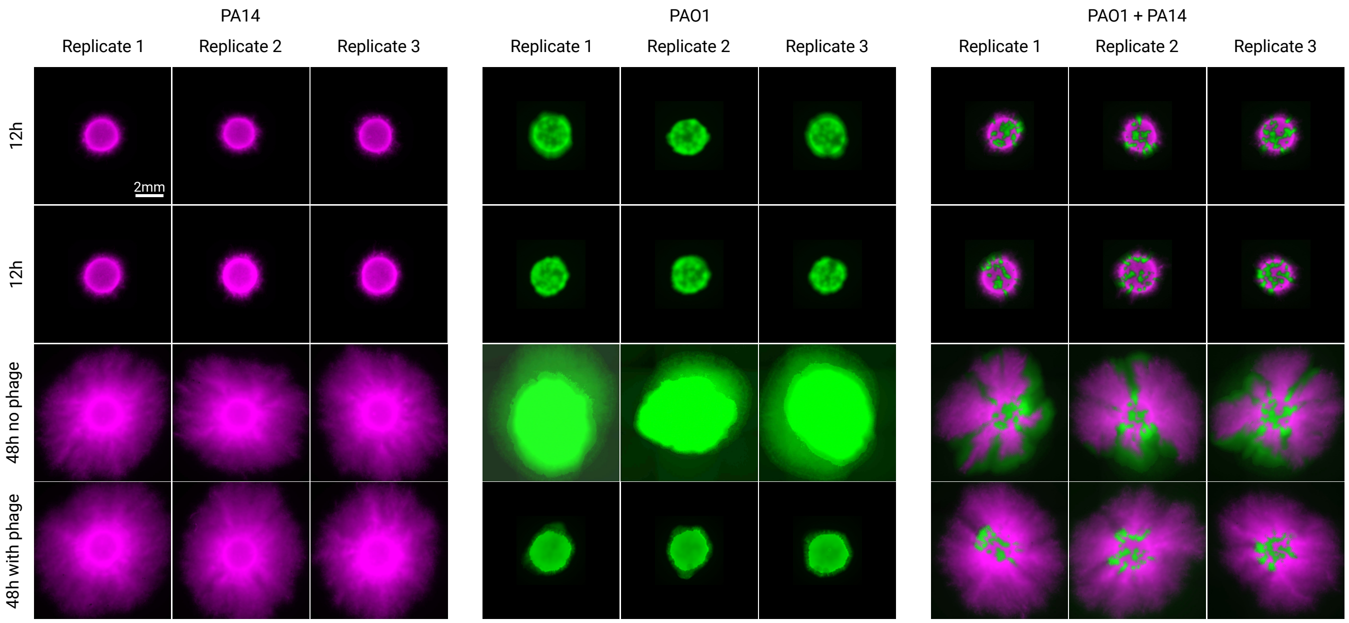

Supplementary Figure 3. Fluorescence microscopy images of colonies at 12 and 48 hours in a preliminary experiment similar to those shown in Fig. 2B. PA14 are tagged with mCherry (purple) and PAO1 with GFP (green). The first row shows the colonies that were transferred to agar without phage, while the second row are the colonies that were later exposed to phage. The first and second row therefore correspond to the images in the third and fourth row, respectively.

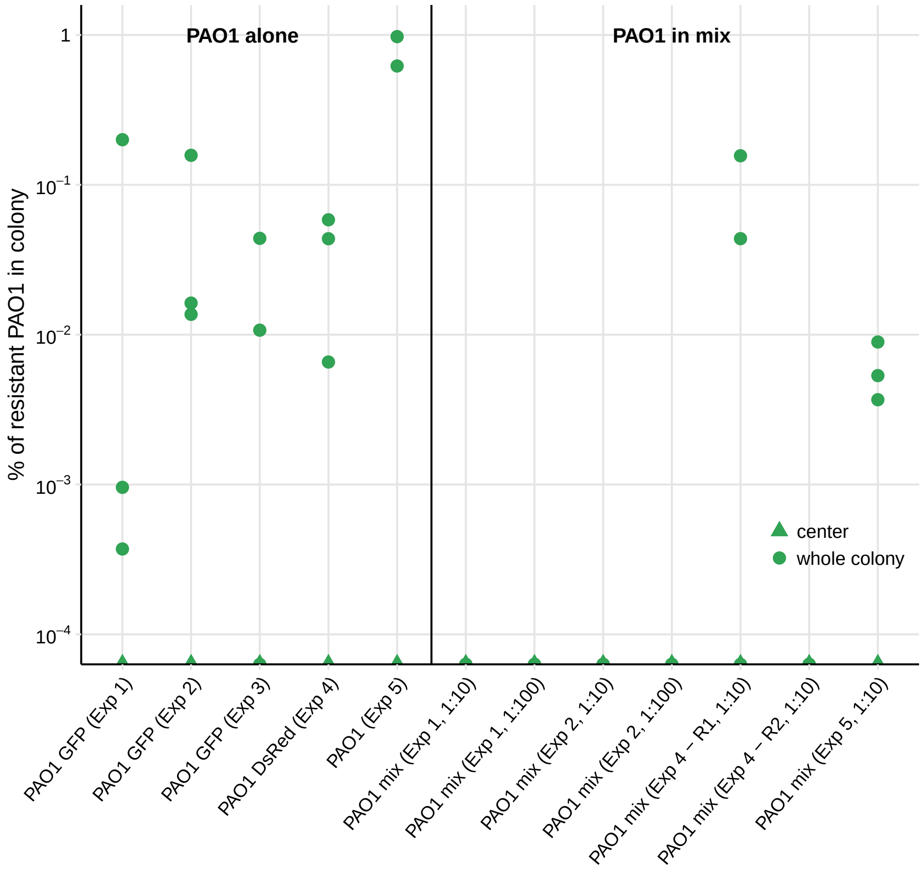

Supplementary Figure 4. Similar to Fig. 2D, but including data from all experiments for comparison. Experiment 1 corresponds to the data in Fig. 2B and 5, experiment 2 to data in Fig. 2C-F and 1, experiment 3 was a repeat of experiment 2 (data not shown), experiment 4 to data shown in Supplementary Figure 14, and experiment 5 was with higher phage dose, corresponding to Supplementary Figure 2. We never found resistance in the center of any colonies. We only found resistance in mixed colonies either if the resistant strain was completely outcompeted by the sensitive one, in which case the colony behaved as a mono-culture (Exp 4 - resistant strain R1); or if the initial dose of phage was 3 orders of magnitude higher, leading to increased selection pressure (Exp 5).

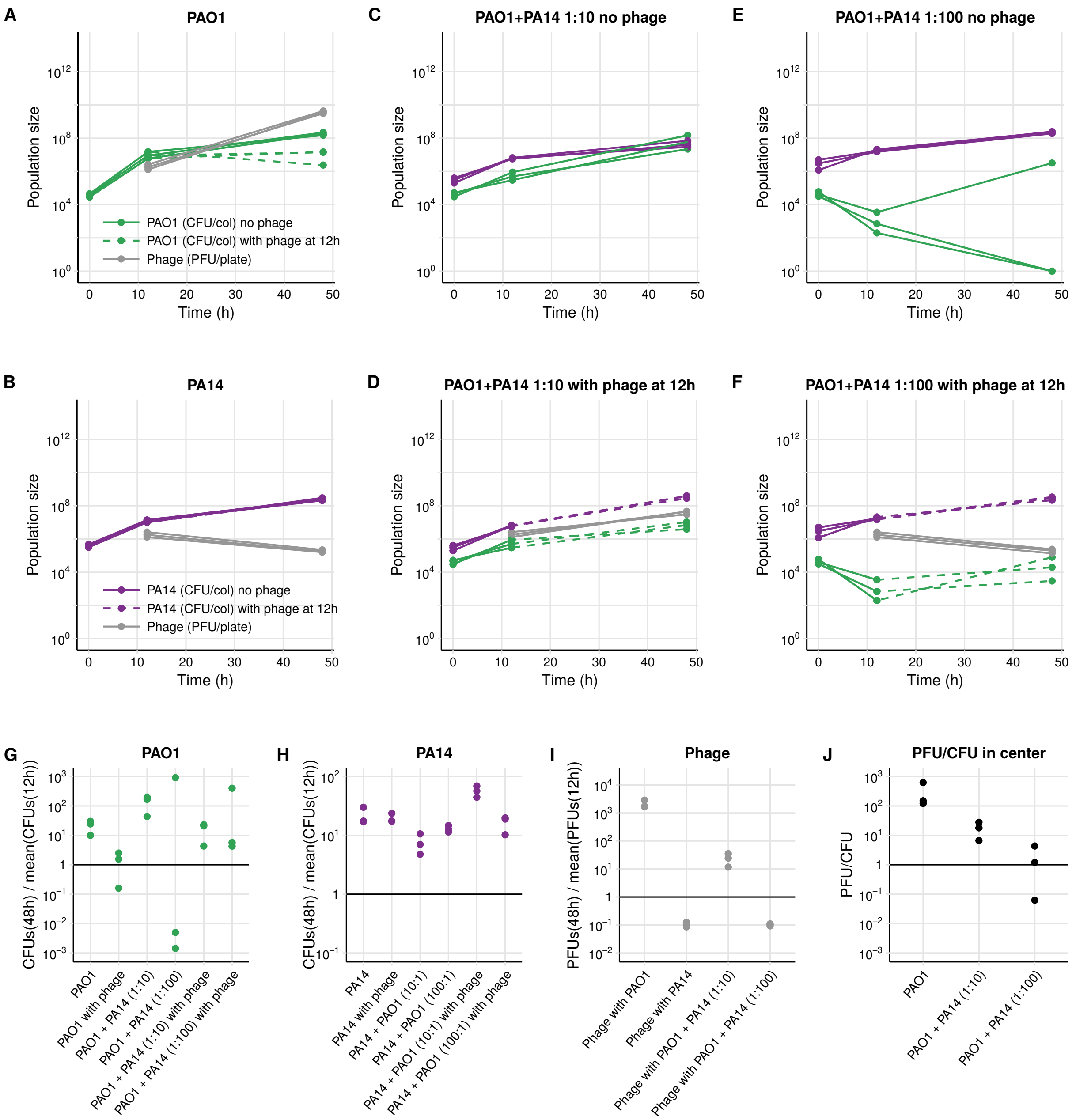

Supplementary Figure 5. Growth curves corresponding to data in Fig. 2B. Here, PA14-mCherry was used, making it harder to quantify population sizes compared to Supplementary Figure 1. (A) Growth curve of PAO1 with and without phage. PAO1 cease to grow following phage exposure. (B) PA14-mCherry with and without phage. No difference was observed following phage treatment, and phage decreased as in a control experiment with no bacteria. (C) Co-culture colony of the two strains at a 1:10 ratio, in the absence of phage. (D) Co-culture colony of the two strains at a 1:10 ratio, with phage, PAO1 again ceases to grow significantly. (E) Same as (C) but with a 1:100 ratio between the two strains (higher initial population size of PA14-mCherry). Due to the increased competition, PAO1 cannot grow as well. (F) Same as (D) but with a 1:100 ratio between the two strains. Again, due to increased competition, there are fewer PAO1 cells, so phage cannot replicate as much. This leads to the lower overall phage population size as shown in Fig. 2F. (G) The ratio of population sizes of PAO1 at 48 and 12 hours in the different colonies. (H) The ratio of population sizes of PA14-mCherry at 48 and 12 hours. (I) The ratio of population sizes of of phage at 48 and 12 hours. (J) To determine whether phage could diffuse into the colonies, we touched the centers with an inoculation loop and counted the PAO1 CFUs and phage PFUs. Results are comparable to Fig. 2E.

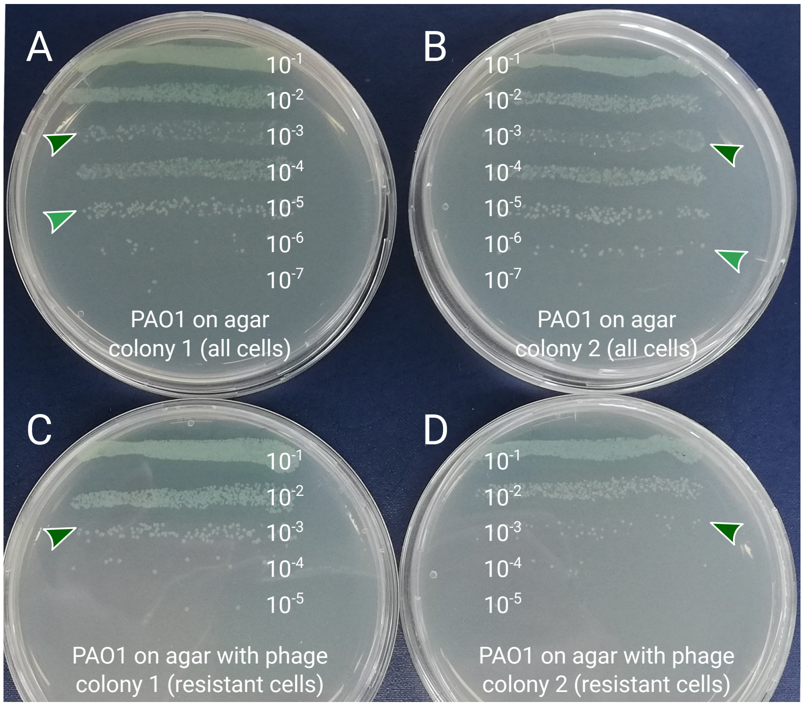

Supplementary Figure 6. Dilutions performed to count CFUs of PAO1 colonies exposed to phage at 12h and then harvested at 48h. The petridishes in the top row (A-C) contained agar only, while those in the bottom row (D-F) were saturated with phage. Each pair of petridishes above one another (e.g. A and C) came from dilutions of the harvested and washed cells of one of two replicate colonies. In each petridish, parallel lines were plated, each from a different dilution as annotated. In plates in A and B, note that colony density does not decrease gradually as one would expect from a dilution series. We think this is because some cells are already infected by phage and lyse once plated onto the agar, killing their sensitive neighbors when at high density. Accordingly, at dilution 10^{-3} (dark green arrowheads in A and B)), the observed cells are likely to mostly be resistant. Indeed, colony density at this dilution corresponds to the dilutions on the agar plates below (dark green arrowheads in C and D), which were saturated with phage, thereby only allowing resistant cells to form colonies. Instead, in the top plates, sensitive cells, which were more abundant, could grow only at higher dilutions (e.g. 10^{-5}, light green arrowheads) where their neighbors were not infected.

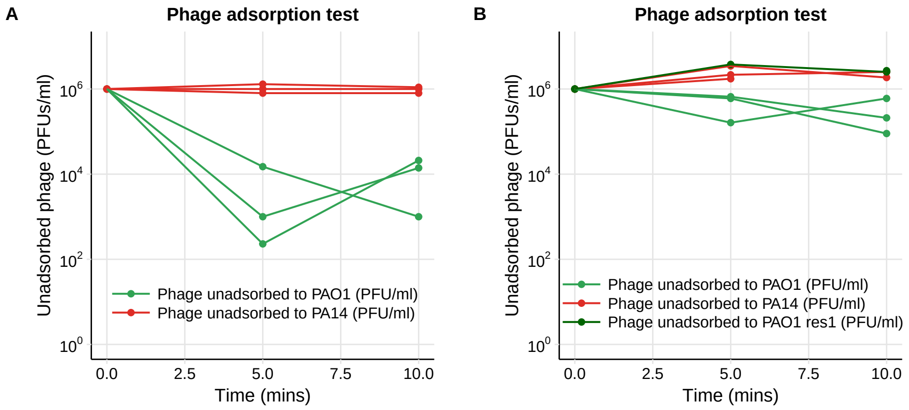

Supplementary Figure 7. Adsorption test. Different strains were mixed with phage for 5 or 10 minutes, then centrifuged and the supernatant used to count PFUs. If no attachment occurred, the phage population size remained the same, whereas phage attachment resulted in a decrease in recovered population size. (A) Comparing PA14 and PAO1. (B) Repeat of the previous assay, in addition to PAO1 resistant strain 2. Initial phage numbers are theoretical based on the preparation of the phage stocks.

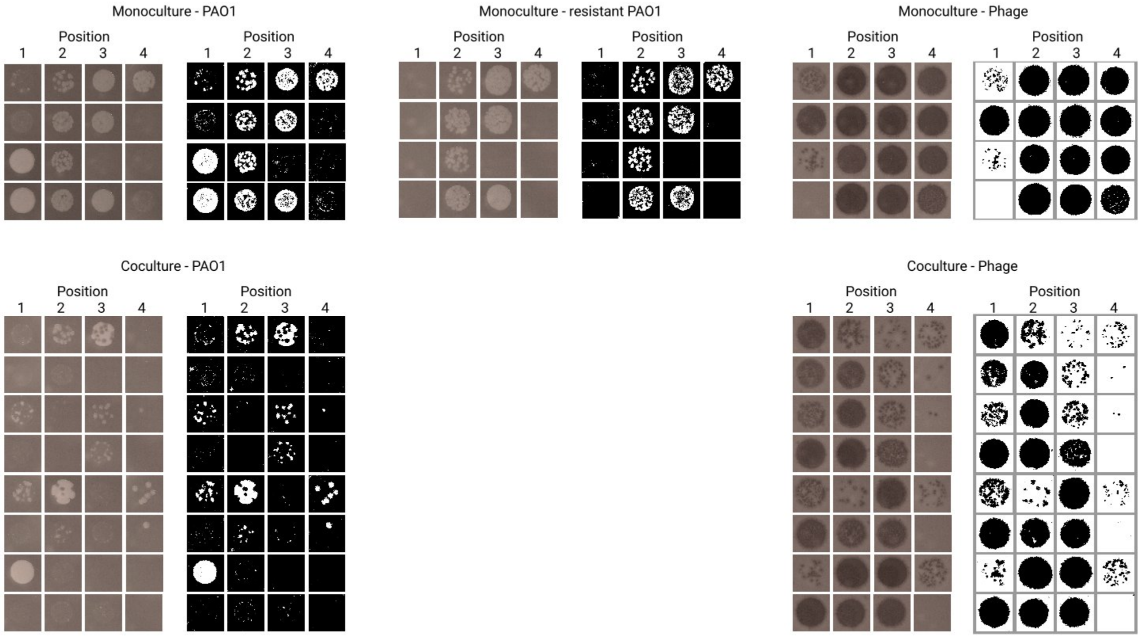

Supplementary Figure 8. Dataset used to generate plot in Supplementary Figure 11. Positions correspond approximately to those touched with the toothpick as shown in Fig. 4A. On the left we show all original images, and to the right the thresholded images (see Methods). These thresholded images are then used to compute the density of bacteria (all bacteria here are PAO1, agar contains gentamicin, which is selective for PAO1) and phage. Rows show different replicate colonies per condition (here: 4 for monoculture and 8 for coculture). Images for resistant PAO1 in the co-culture condition are not shown since nothing grew. The code to apply the threshold was written in Matlab® R2017b (using the image processing toolbox) and is available upon request.

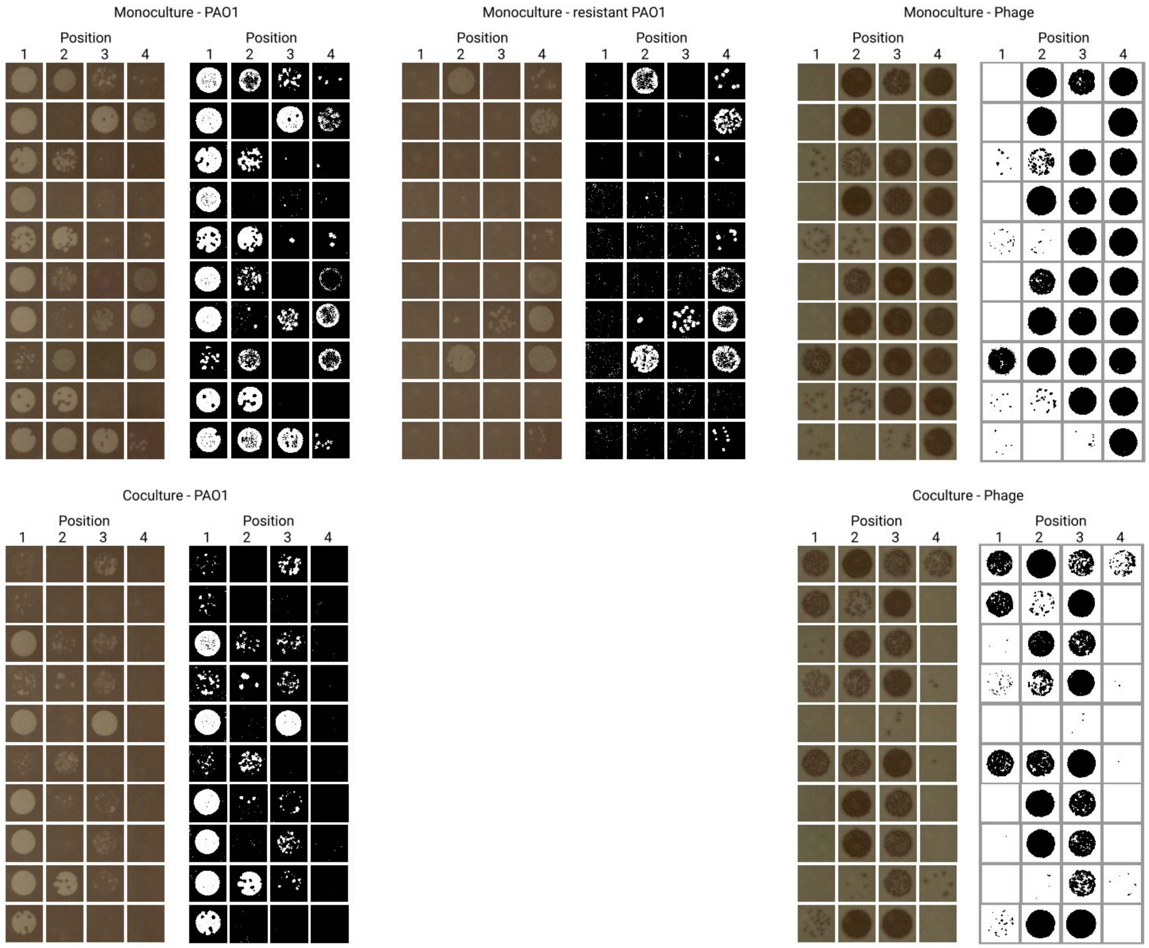

Supplementary Figure 9. Dataset used to generate plot in Fig. 4. Positions correspond approximately to those touched with the toothpick as shown in Fig. 4A. On the left we show all original images, and to the right the thresholded images (see Methods). These thresholded images are then used to compute the density of bacteria (all bacteria here are PAO1, agar contains gentamicin, which is selective for PAO1) and phage. Rows show different replicate colonies per condition (here: 10 for monoculture and 10 for coculture). Images for resistant PAO1 in the co-culture condition are not shown since nothing grew. The code to apply the threshold was written in Matlab® R2017b (using the image processing toolbox) and is available upon request.

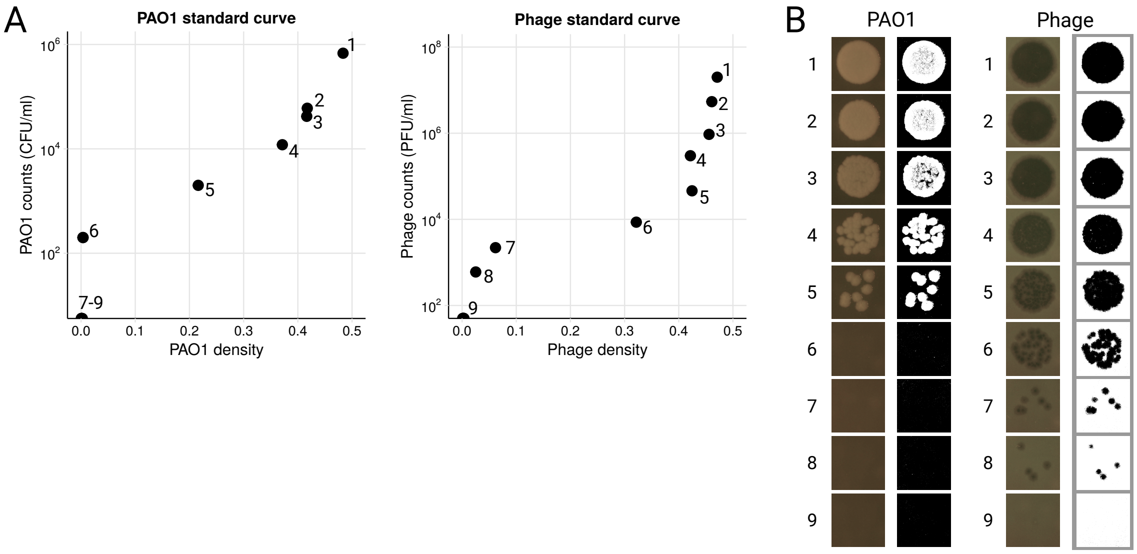

Supplementary Figure 10. Standard curve showing how density maps to population size. (A) PAO1 density (left) and phage density (right) according to our measurement method versus CFU/ml and PFU/ml, respectively. The number next to each point shows how it corresponds to the images in panel B. (B) The images used to generate the data in panel A. Note that this approach allows us to assess variations between ~10^2 and ~10^5 CFU/ml, while phage densities can be distinguished between ~10^3 and ~10^5. The approach does not have a very high resolution, but is much quicker than counting CFUs and PFUs and can be used as a complementary method.

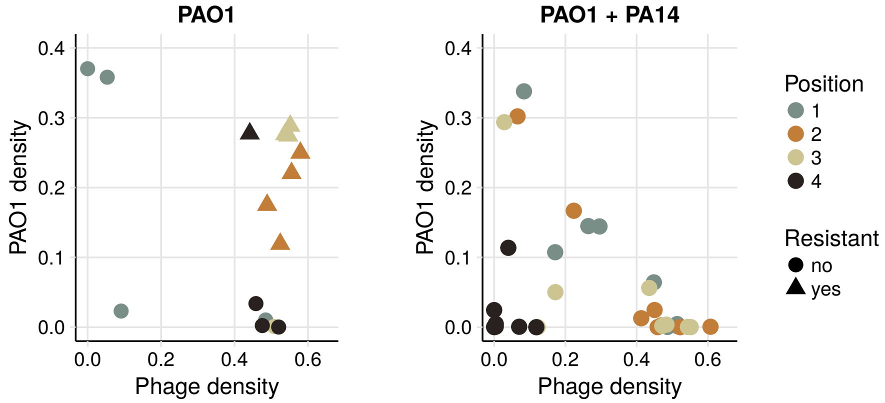

Supplementary Figure 11. Sampling colonies to determine co-occurrence patterns of phage and bacteria (repeat of experiment shown in Fig. 4). Each dot or triangle corresponds to a sample in one position in one colony. The left and right panels show samples taken from 4 PAO1 (16 points) and 8 mixed colonies (32 points). Resistance was determined by similarly thresholding images of drops grown on LB agar with gentamicin and saturated with ~10^{10} phage (see Supplementary Figure 8 for the full data set). The different colors represent the positions sampled as shown in Fig. 4A.

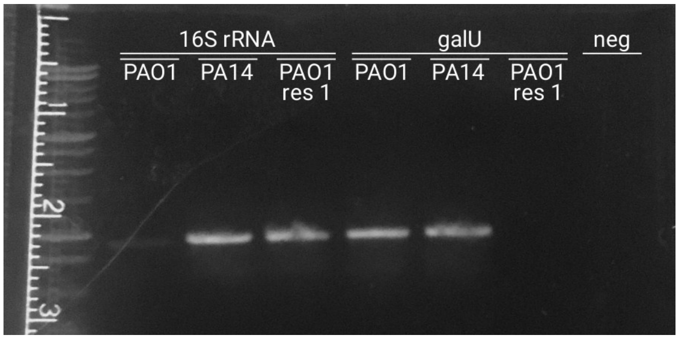

Supplementary Figure 12. Photo of gel electrophoresis where we ran the PCR product of the amplified 16S gene of PAO1, PA14 and PAO1 resistant strain 1, and of the amplified galU gene of the same three strains. This shows that the galU gene is absent in the resistant strain. We used GIMP to convert the image to grayscale and Inkscape to add the annotation of the different lanes.

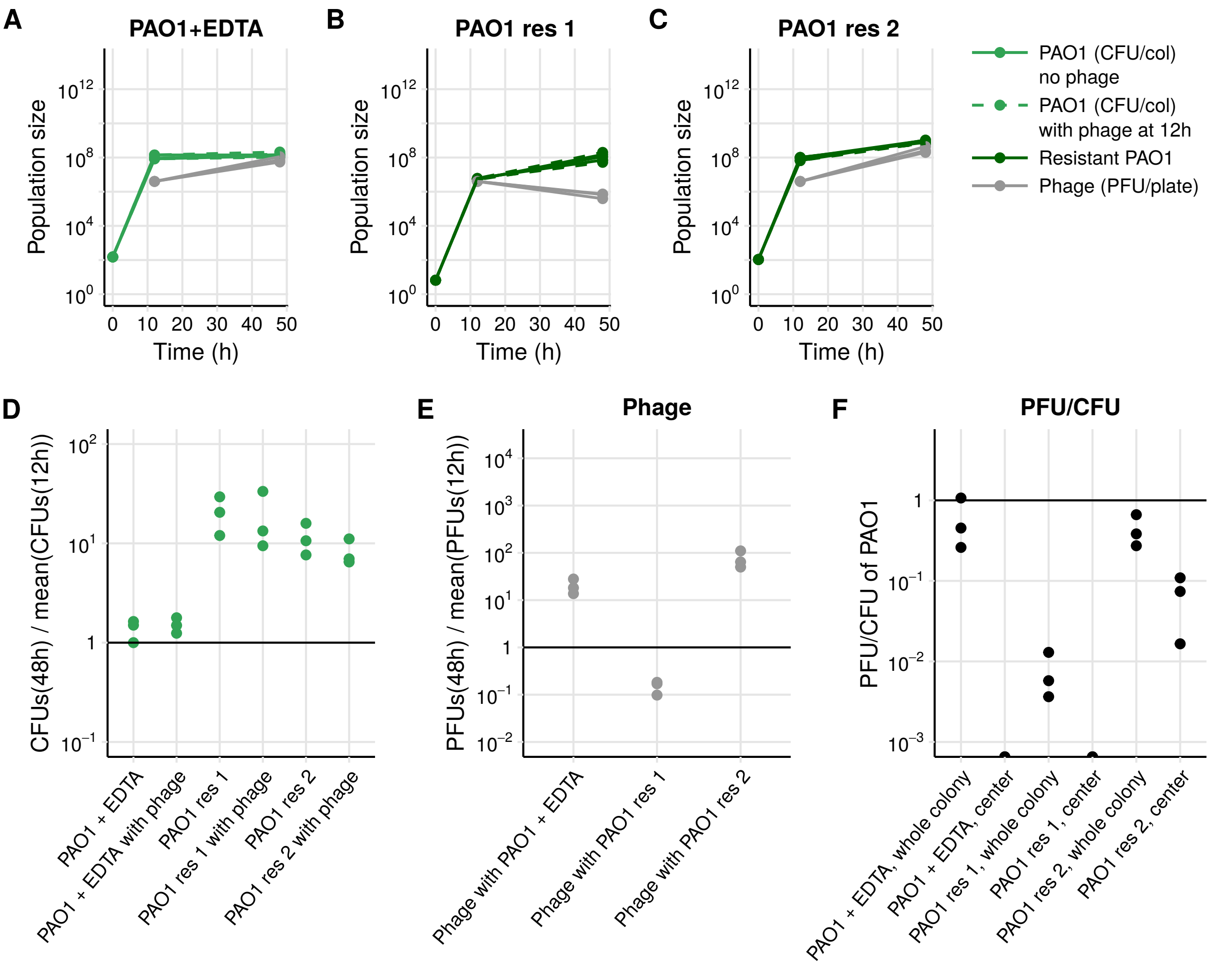

Supplementary Figure 13. Experiment with mono-cultures of the wildtype PAO1 growing on EDTA and two resistant PAO1 isolates growing on LB. (A) Growth curve of PAO1 on EDTA with and without phage. Even though the phage replicates somewhat, there is little growth nor death. (B) The first resistant isolate (res 1), studied in more detail in the main text, is completely resistant, such that the phage decrease as in Fig. 2C with phage alone, or with PA14. (C) The second resistant strain (res 2) is only partially resistant (approx. 1 in 10 CFUs were able to form colonies on phage-saturated agar plates), such that the phage can still replicate. (D) The ratio of population sizes of bacteria at 48 and 12 hours. (E) The ratio of population sizes of phage at 48 and 12 hours. (F) To determine whether phage could diffuse into the colonies, we touched the centers with an inoculation loop and counted the PAO1 CFUs and phage PFUs. No phage were detected in the centers of colonies growing on EDTA or completely resistant colonies. All colonies contained fewer phage than in the data shown in Fig. 2E. Some of these data are also shown in Fig. 5, but included here again for comparison with resistant strain 2.

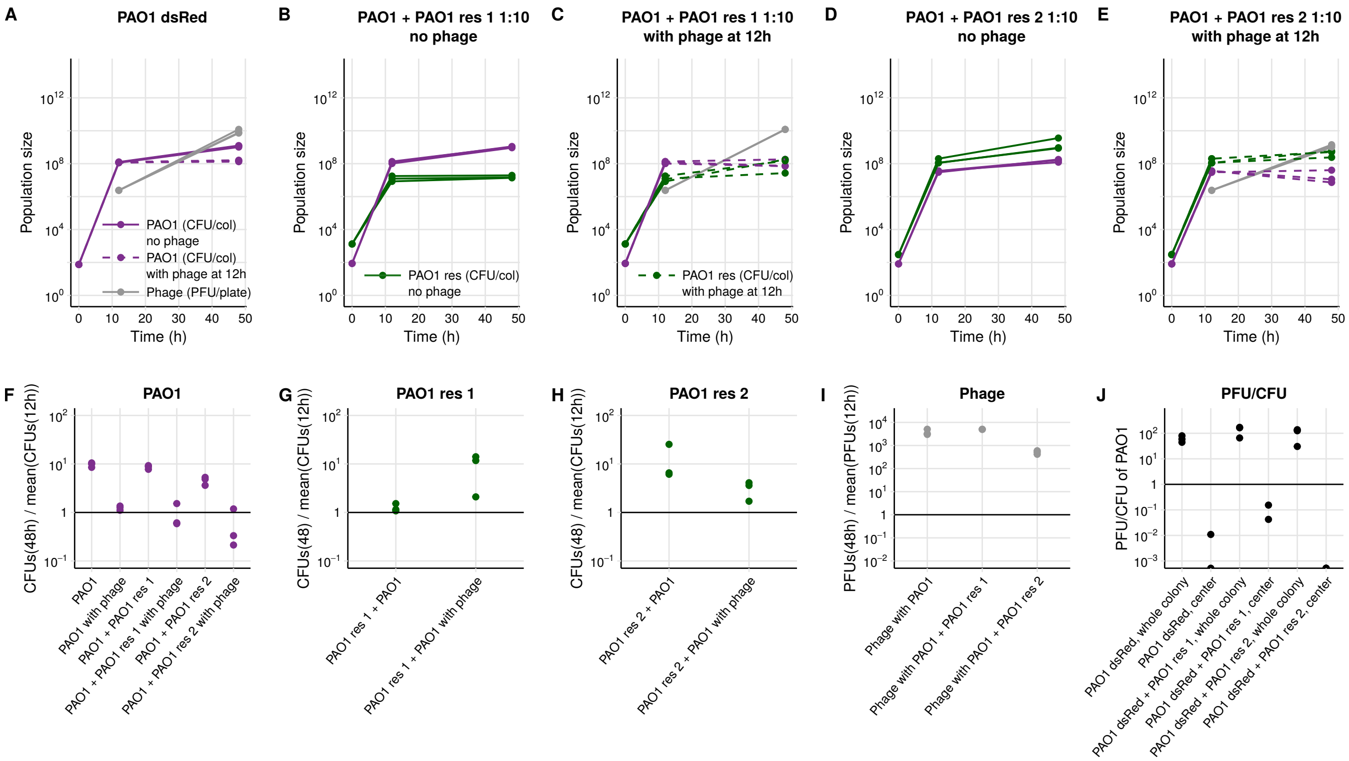

Supplementary Figure 14. Experiment 4 where we replaced PA14 with one of two resistant PAO1 isolates (resistant strain 1 (galU mutant, Supplementary Figure 12) which is fully resistant, and resistant strain 2 which is partially resistant (Supplementary Figure 13B, C)). To distinguish the two strains when in co-culture colonies, we used a wildtype PAO1 strain with a DsRed tag. The resistant strains were labelled with GFP. (A) Growth curve of PAO1-DsRed with and without phage, comparable to Supplementary Figure 1. (B) Growth curve of PAO1-DsRed when in co-culture with resistant strain 1, with no phage. The fitness cost to becoming completely resistant to phage is visible in its slow growth. (C) Same as (B) but with phage. Here, the phage infects and replicates similar to the case where wildtype PAO1 is in mono-culture. (D) Same as (B) but with resistant strain 2. (E) Same as (D) but with phage. (F) The ratio of population sizes of PAO1-DsRed at 48 and 12 hours in the different colonies. (G) The ratio of population sizes of PAO1 resistant strain 1 at 48 and 12 hours. (H) The ratio of population sizes of PAO1 resistant strain 2 at 48 and 12 hours. (I) The ratio of population sizes of phage at 48 and 12 hours. (J) To determine whether phage could diffuse into the colonies, we touched the centers with an inoculation loop and counted the PAO1 CFUs and phage PFUs. All three treatments behave similar to PAO1-GFP mono-cultures (Fig. 2E), PAO1-DsRed because it is isogenic, with resistant strain 1 because it grows so little, leaving PAO1-DsRed to dominate as in mono-culture, and resistant strain 2 because it can also be infected, so there are many phage. Some of these data are also shown in Fig. 5, but included here again for comparison with resistant strain 2.

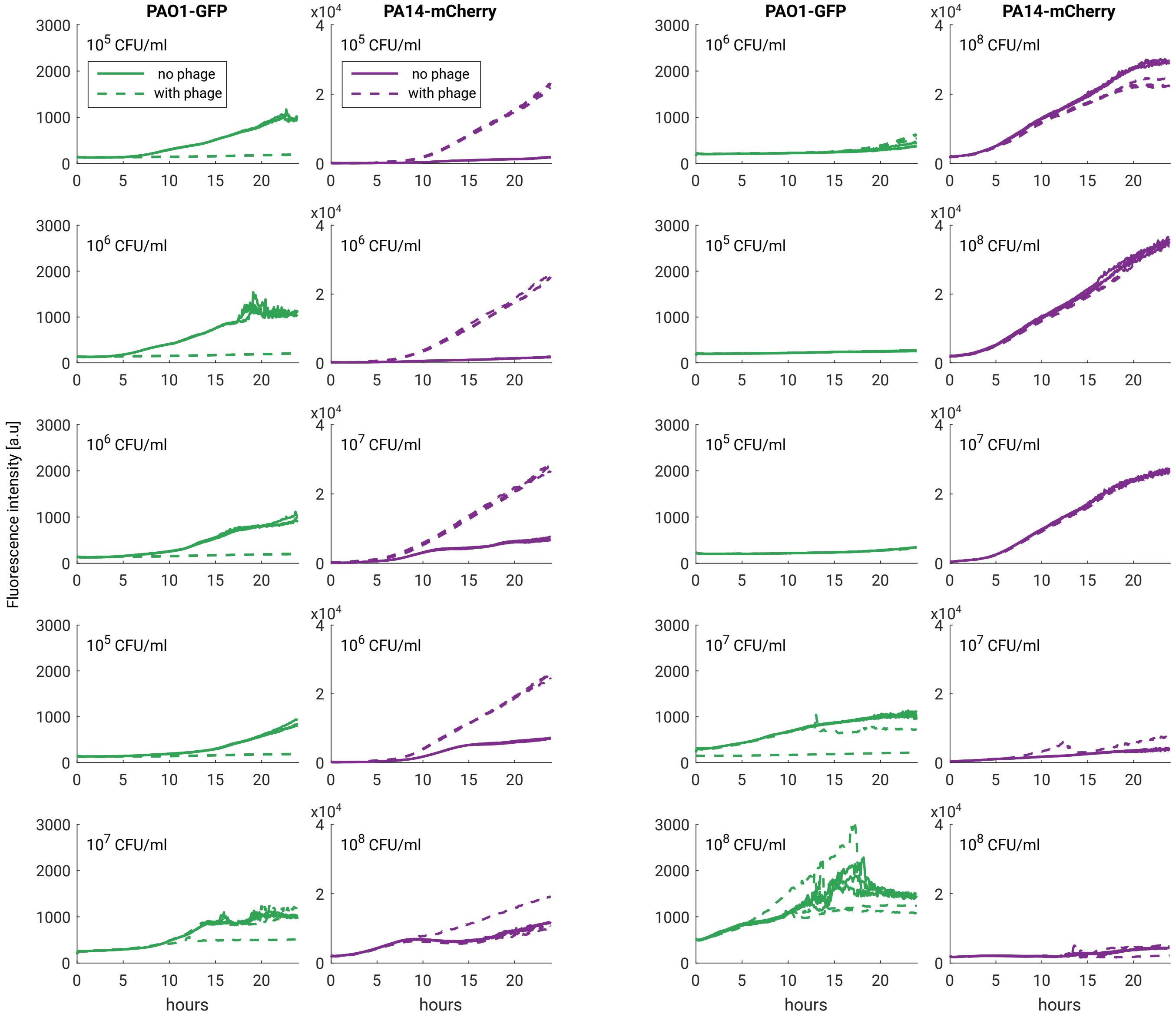

Supplementary Figure 15. Data from a pilot study to decide on ratio of PAO1 and PA14 to grow in liquid co-culture. Each two adjacent panels show measurements from the same wells, with the left panel measuring the GFP signal (to quantify PAO1 growth) and the right panel the mCherry signal (to quantify PA14 growth). Units are arbitrary. Solid lines show wells that contained no phage, while dashed lines contained phage, added in a titer identical to the initial CFU/ml of PAO1 (MOI=1). All conditions were repeated in triplicate. The initial population size of PAO1 and PA14 are written on the top left of each panel. When cell numbers became too abundant, the fluorescence intensity became very noisy (e.g. panel with 10^8 initial cells of PAO1). We chose conditions where both PAO1 and PA14 could grow well in each other's presence without phage. This condition was where PAO1 started with 10^6 CFU/ml and PA14 with 10^8 CFU/ml (plot pair in top right).

Microscopy equipment and settings. Colony images in Fig. 2B were taken on two different days (after 12h and 48h) by directly placing the colonies under an upright microscope (no coverslips were used). All images have a resolution of 312 pixels per cm (pixel size: 0.0032×0.0032 cm^2), and a bit depth of 32 per pixel (RGB). We used a GFP filter set (excitation: 470/40, emission: 525/50) and a mCherry filter set (excitation: 545/30, emission: 620/60), as described in the Methods section. Image brightness and contrast were adjusted automatically by Visiview® 4.1.0 software. Overlaying of the GFP and mCherry channels was done in Visiview® 4.1.0 using pseudocolors green for GFP and red for mCherry.

Images taken at 12h were taken in one single shot using the 2.5x objective as described in the Methods section. Mono-culture PAO1 colonies had a GFP exposure time of 6ms, while mixed colonies were exposed to GFP for 20ms. All colonies containing PA14 were exposed to mCherry for 6ms. Magnification was 0.75.

Images taken at 48h were assembled from multiple shots because the colonies were too large to fit on a single image. Due to problems with the microscope, 3 vertical image series were taken (each composed of 3 shots that were combined using Visiview® 4.1.0) and then stitched together using autostitch software as described in the Methods section. Magnification was 0.33. Exposure times are listed in Supplementary Table 1.

Treatment	Channel	Exposure time
PAO1 no phage	GFP	393 - 425ms
PAO1 with phage	GFP	17ms
PA14 (no/with) phage	mCherry	10ms
PAO1 + PA14 no phage	GFP	69-234ms
PAO1 + PA14 no phage	mCherry	10ms
PAO1 + PA14 with phage	GFP	91ms
PAO1 + PA14 with phage	mCherry	10ms

Supplementary Table 1. Exposure times for colony images in Fig. 2B at 48h. Where a range of exposure times is shown, different images were taken with different exposure times due to the use of automatic exposure, and the resulting images stitched together.
